# From early lessons to new frontiers: the worm as a treasure trove of small RNA biology

**DOI:** 10.3389/fgene.2014.00416

**Published:** 2014-11-27

**Authors:** Elaine M. Youngman, Julie M. Claycomb

**Affiliations:** ^1^Department of Biology, Villanova UniversityVillanova, PA, USA; ^2^Department of Molecular Genetics, University of TorontoToronto, ON, Canada

**Keywords:** *C. elegans*, small RNA pathways, Argonaute proteins, gene expression regulation, gene silencing, post-transcriptional regulation of gene expression, RNAa, RNAi

## Abstract

In the past 20 years, the tiny soil nematode *Caenorhabditis elegans* has provided critical insights into our understanding of the breadth of small RNA-mediated gene regulatory activities. The first microRNA was identified in *C. elegans* in 1993, and the understanding that dsRNA was the driving force behind RNA-mediated gene silencing came from experiments performed in *C. elegans* in 1998. Likewise, early genetic screens in *C. elegans* for factors involved in RNA interference pointed to conserved mechanisms for small RNA-mediated gene silencing pathways, placing the worm squarely among the founding fathers of a now extensive field of molecular biology. Today, the worm continues to be at the forefront of ground-breaking insight into small RNA-mediated biology. Recent studies have revealed with increasing mechanistic clarity that *C. elegans* possesses an extensive nuclear small RNA regulatory network that encompasses not only gene silencing but also gene activating roles. Further, a portrait is emerging whereby small RNA pathways play key roles in integrating responses to environmental stimuli and transmitting epigenetic information about such responses from one generation to the next. Here we discuss endogenous small RNA pathways in *C. elegans* and the insight worm biology has provided into the mechanisms employed by these pathways. We touch on the increasingly spectacular diversity of small RNA biogenesis and function, and discuss the relevance of lessons learned in the worm for human biology.

## THE WORM, FROM HUMBLE BEGINNINGS TO KEY CONTRIBUTIONS

Although *Caenorhabditis elegans* is a simple organism in many ways, the worm has been a keystone of several conserved areas of biological exploration, including apoptosis, neurobiology, the establishment of developmental axes, and small RNA-mediated gene regulation (Riddle et al., 1997). *C. elegans* emerged as a model organism nearly 50 years ago when Brenner (1974) chose *C. elegans* instead of *C. briggsae* or other candidate nematode species. In a stroke of luck that Brenner (1974) could not have foreseen, his serendipitous choice in model organisms, enabled the later discovery of RNA interference (RNAi) and endogenous small RNA pathways, including microRNAs (miRNAs), ultimately transforming molecular biology and our understanding of gene regulation.

Small RNA-mediated gene regulatory pathways are characterized by two main components: small RNAs, which provide sequence specificity in selecting target transcripts, and Argonaute proteins which coordinate both small RNA and complementary target RNA to regulate gene expression (**Table 1**; Czech and Hannon, 2011). These pathways can act post-transcriptionally (generally in the cytoplasm) or co-transcriptionally (in the nucleus), and while most known functions of these pathways relate to silencing gene expression, a few functions for small RNA pathways in licensing or promoting gene expression have been also described (see below; Ketting, 2011b). Small RNAs are generally derived from double-stranded RNA (dsRNA), although the sources of endogenous dsRNA vary between pathways. Early investigation into the process of RNA-mediated interference (Rocheleau et al., 1997; now known as exogenous RNAi or exo-RNAi) in *C. elegans* led to the key identification of dsRNA – and not antisense RNA, as was initially suspected – as the active agent for gene silencing (Fire et al., 1998). In these studies, for which Mello and Fire were awarded the Nobel prize, the authors built on previous observations from Guo, Kemphues, and others to show that neither purified antisense nor sense RNA with homology to the *unc-22* gene was alone capable of silencing *unc-22*. Instead, it was only dsRNA with homology to the target gene that could silence expression (Guo and Kemphues, 1995; Fire et al., 1998). Subsequent studies in several organisms showed that dsRNA was processed by the endonuclease Dicer into small RNAs (18–22 nt) known as short interfering RNAs (siRNAs; Bernstein et al., 2001; Ketting et al., 2001; Knight and Bass, 2001). Although siRNAs are produced as a duplex, only one strand, which provides sequence specificity to Argonautes during gene regulation, is loaded onto the Argonaute (Martinez et al., 2002).

**Table 1:** Summary of *C. elegans* small RNA pathways discussed in this review (see text for references).

Small RNA pathway		Biosynthetic enzyme(s)	5′ signature	3′ signature	Associated AGO(s)	Expression	Subcellular compartment(s)	Targets	Regulatory outcome
miRNA		RNA PolII; DCR-1; DRSH-1	pU	Hydroxyl	ALG-1, -2	Widespread	Cytoplasm	Diverse genes	Post-transcriptional repression
Exogenous RNAi	Primary siRNAs	DCR-1	p	Hydroxyl	RDE-1	Widespread	Cytoplasm	Exo-RNAi targets	
	Secondary siRNAs (22G RNAs)	RRF-1/EGO-1 RdRP modules	pppG	Hydroxyl	WAGOs	AGO-specific	Nucleus (NRDE-3, HRDE-1) Cytoplasmic (other WAGOs)	RDE-1 targets	Silencing (TGS; PTGS)
piRNA	21U-RNA	RNA PolII	pU	2′-*O*-methyl	PRG-1, -2	Germline	P granules	Transgenes, transposons, pseudogenes	
	WAGO-class 22G RNAs	RRF-1/EGO-1 RdRP modules	pppG	Hydroxyl	WAGOs	Germline-enriched	Nucleus (HRDE-1) P granules (other WAGOs)	PRG-1 targets	Silencing (TGS; PTGS)
Embryo 26G	26G RNAs	RRF-3; DCR-1	pG	2′-*O*-methyl	ERGO-1	Germline, embryo		Duplicated genes, non-coding loci	
	WAGO-class 22G RNAs	RRF-1/EGO-1 RdRP modules	pppG	Hydroxyl	WAGOs	Germline-enriched	P granules	ERGO-1 targets	Silencing (PTGS)
Spermatogenesis 26G	26G RNAs	RRF-3; DCR-1	pG	Hydroxyl	ALG-3, -4	Spermatogenesis	P granules	Spermatogenesis-enriched genes	
	CSR-1-class 22G RNAs	EGO-1 RdRP module	pppG	Hydroxyl	CSR-1	Germline	P granules, nucleus	Subset of ALG-3, -4 targets	Transcriptional licensing
	WAGO-class 22G RNAs	RRF-1/EGO-1 RdRP modules	pppG	Hydroxyl	WAGOs	Germline-enriched	P granules	Subset of ALG-3, -4 targets	Silencing (PTGS)
CSR-1	CSR-1-class 22G RNAs	EGO-1 RdRP module	pppG	Hydroxyl	CSR-1	Germline	P granules, nucleus	Germline-expressed genes	Transcriptional licensing

*This table is not intended to be an exhaustive compilation but rather an overview to accompany the material highlighted in the text. p, monophosphate, ppp, triphosphate, TGS, transcriptional gene silencing, PTGS, post-transcriptional gene silencing.*

*Caenorhabditis elegans* can raise a robust silencing response to exogenously provided dsRNA in a process known as environmental RNAi. In the lab, environmental RNAi is generally mediated by soaking worms in a dsRNA solution or by feeding the worms *E. coli* that express dsRNA (Whangbo and Hunter, 2008). This response can then spread throughout the worm in a process known as systemic RNAi to silence genes in cells distant from the initial site of dsRNA uptake. Notably, Brenner’s (1974) abandoned alternate species, *C. briggsae*, along with a number of other related nematode species, cannot initiate an environmental RNAi response. This defect is in spite of being able to perform systemic RNAi (Winston et al., 2007; Nuez and Felix, 2012), and is due to differences in the function of the dsRNA transporter *sid-2*. Notably, when expressed from a transgene in *C. briggsae*, *C. elegans-sid-2* is sufficient to restore the ability to take up dsRNA from the environment and silence gene expression (Winston et al., 2007).

Just as the identification of dsRNA as the agent of RNAi facilitated the genetic manipulation of previously intractable model systems such as cell culture, the ability of *C. elegans* to respond to environmental RNAi allowed a new era of genetic screening in the worm. Collections of arrayed bacteria expressing dsRNA for the vast majority of protein coding genes have been generated and have made countless reverse genetic screens possible, akin to the lentiviral dsRNA libraries that enable genome-wide screening in cell culture (Kamath et al., 2003; Moffat et al., 2006). Likewise, the ability to assay for resistance to environmental RNAi has enabled forward genetic screens to identify the factors involved in endogenous small RNA pathways. For instance, clever initial screens for mutants that could not silence the essential gene *pos-1* (and thus survived when all other worms died due to loss of *pos-1*) were successful in identifying the first known *C. elegans* Argonaute RDE-1 (RNAi DEficient) and other factors necessary for exo-RNAi (Tabara et al., 1999). Thus, Brenner’s (1974) initial choice of *C. elegans* as a model system clearly altered the trajectory of RNAi research in a positive manner.

## THE DISCOVERY OF microRNAs FORESHADOWS AN ENDO-siRNA DELUGE

Prior to the appreciation that exogenously provided dsRNA resulted in the silencing of complementary target transcripts, the first miRNA, *lin-4*, was identified by genetic analysis in the worm. Initially, *lin-4* was identified as a genetic suppressor of developmental timing defects brought about by mutation of the protein coding gene, *lin-14* (Lee et al., 1993; Wightman et al., 1993). Confoundingly, the locus encoding *lin-4* did not appear to be capable of encoding a protein, but instead the authors identified a small RNA species produced from the locus. This small RNA possessed complementarity to the *lin-14* transcript, and in fact, *lin-14* levels decreased when *lin-4* was expressed. Thus, the authors proposed an antisense mechanism for *lin-4* activity on *lin-14*, which we now know to be correct. Notably, the *lin-4* sequence appeared to be conserved among several species of nematodes, opening the possibility that antisense regulation of mRNAs by small RNAs may be a widespread gene regulatory mechanism (Lee et al., 1993; Wightman et al., 1993). Subsequent identification of additional miRNAs revealed broad conservation (Lau et al., 2001; Lagos-Quintana et al., 2001; Lee and Ambros, 2001). Today, miRNAs are the most widely studied small RNA species, and have been implicated in human diseases ranging from cancer to cardiovascular disease, and in the development and differentiation of tissues in organisms ranging from plants to humans (Ketting, 2011a).

**FIGURE 1:**
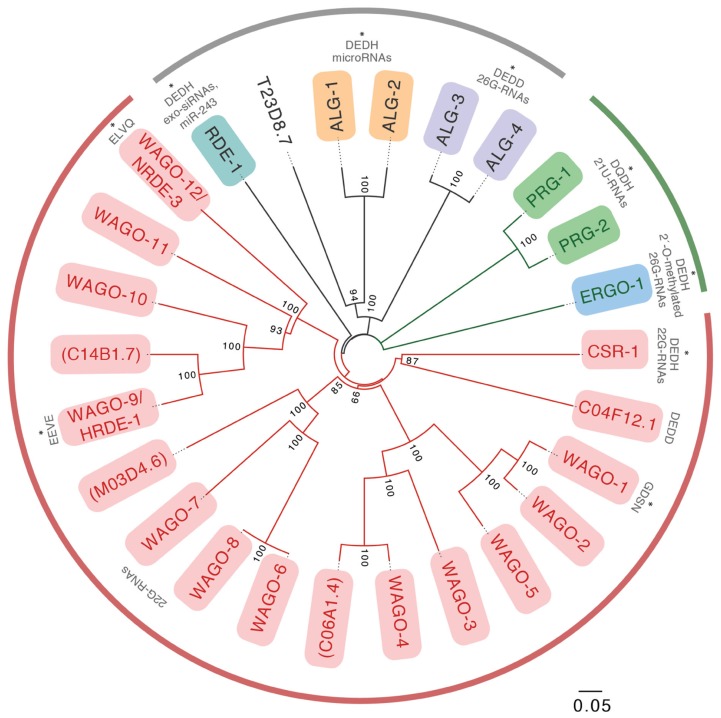
**A phylogenetic tree of the full length *Caenorhabditis elegans* Argonaute proteins.** Members of the WAGO subclade are shown in red lines, members of the PIWI subclade are shown in green lines, and members of the Argonaute subclade are shown in black lines. Putative pseudogenes are denoted with parentheses. Those AGOs for which small RNA profiles were studied previously are marked with an asterisk. All others have yet to be examined. Residues present at the catalytic tetrad site are denoted for each of the AGOs previously examined and for those additional AGOs possessing a putative functional catalytic site (i.e., C04F12.1). Full-length protein sequences were obtained from Wormbase.org. Multiple sequence alignment was generated using T-coffee and bootstrapping was conducted using ClustalX. Numbers on the branches report the level of confidence by bootstrap analysis (1000 bootstrap replicates). Bootstrap values <70% were excluded from the tree.

We know today that miRNAs represented only the tip of the iceberg. 15 years of research have revealed that at least three endogenous small RNA pathways in addition to miRNAs operate in *C. elegans*: 22G-RNAs, 26-RNAs, and piRNAs/21U-RNAs (**Table 1**; Billi et al., 2014). While we will discuss key features of these pathways below, we refer you to Billi et al. (2014) for more comprehensive coverage. The activities of these pathways are orchestrated by as many as 27 Argonaute effectors, which is in sharp contrast to the 10 Argonaute superfamily proteins in plants, or the mere eight family members in humans (Yigit et al., 2006; Hutvagner and Simard, 2008). The 27 members of the Argonaute superfamily are broken into the Argonaute clade (conserved from plants to humans), the PIWI clade (generally conserved in animals) and an expanded clade of Argonautes that are specific to nematodes (WAGOs; **Figure 1**; Yigit et al., 2006). Although the functions and small RNA binding complements of a handful of Argonautes have been studied in the worm, many family members have yet to be fully characterized.

MicroRNAs are genomically encoded and form hairpin precursors, which are subsequently processed in a stepwise manner by two endonucleases: Drosha (in the nucleus) and Dicer (in the cytoplasm; Ketting, 2011a). While the biogenesis of miRNAs is conserved between species, the biogenesis of other small RNA species varies between organisms. One notable feature of *C. elegans*, plants, and fungi is the presence of RNA dependent RNA polymerase enzymes (RdRPs), which synthesize particular classes of small RNAs in the worm using target transcripts as a template (Smardon et al., 2000; Aoki et al., 2007). This aspect of worm small RNA biology enables the amplification of small RNAs, such that only a few initial Dicer-dependent small RNA molecules can set off a cascade of small RNA production by RdRPs (Sijen et al., 2001). Furthermore, loading of different types of small RNAs at different steps in the amplification cascade onto distinct Argonautes leads to functional diversity. In general, we will refer to small RNAs produced by Dicer as primary siRNAs and those produced by RdRPs as secondary siRNAs throughout this review.

PIWI-interacting RNAs (piRNAs), bound by PIWI-clade Argonautes, are germline-expressed small RNAs that function in genome defense and germ cell development in diverse metazoans (Luteijn and Ketting, 2013). In *C. elegans*, the piRNAs (also called 21U-RNAs, and bearing a 5′ mono-phosphorylated uridine), are generated by two distinct mechanisms, and act as upstream triggers for synthesis by RdRPs of 22G-RNAs that silence target transcripts at both the transcriptional and post-transcriptional levels. One set, the canonical piRNAs, are transcribed by RNA Polymerase II from clusters of piRNA loci on chromosome IV (Ruby et al., 2006; Batista et al., 2008; Das et al., 2008; Billi et al., 2013). Another recently discovered set of piRNAs are generated from abortive transcription of protein coding genes (Gu et al., 2012). Both types of piRNAs bind the PIWI-type Argonaute PRG-1 (Batista et al., 2008; Das et al., 2008; Wang and Reinke, 2008; Gu et al., 2012). piRNAs represent 10s of 1000s of distinct sequences and are thought to pair with their targets using incomplete complementarity (Bagijn et al., 2012; Lee et al., 2012; Shirayama et al., 2012). Owing to this sequence diversity and low specificity in choosing target transcripts, piRNAs play a role in cellular adaptive immunity, combating foreign and deleterious nucleic acid (such as transgenes) for silencing. A subset of protein coding genes are endogenous targets of the piRNA pathway, where targets recognized by piRNA/PRG-1 complexes induce the synthesis of 22G-RNAs. These 22G-RNAs associate with WAGO-1, WAGO-9/HRDE-1, and WAGO-10 to direct downstream silencing, both post-transcriptionally (WAGO-1) and co-transcriptionally (WAGO-9/HRDE-1; Ashe et al., 2012; Bagijn et al., 2012; Buckley et al., 2012; Kamminga et al., 2012; Lee et al., 2012; Shirayama et al., 2012). This facet of the pathway leads to an amplification of the silencing signal and serves a potent adaptive cellular immune response, with silencing occurring at multiple levels in the “lifecycle” of a transcript.

Three RdRP enzymes in the worm (a fourth RdRP does not seem to affect small RNA populations) produce the 22G-RNAs and 26G-RNAs (22 or 26 nucleotides on average, respectively, and possessing a 5′ guanine). The 26G-RNAs are synthesized by the RdRP RRF-3, and are thought to be processed in some manner by Dicer, as 26G-RNAs are depleted in *dcr-1* mutants and bear some characteristics of Dicer processing (such as a 5′ mono-phosphate; Duchaine et al., 2006; Ruby et al., 2006; Gent et al., 2009; Han et al., 2009; Pavelec et al., 2009; Conine et al., 2010; Gent et al., 2010; Vasale et al., 2010). The 26G-RNAs are broken into two categories based on their developmental timing of expression and loading onto particular Argonautes. A subset of 26G-RNAs are modified at the 3′ end by the conserved methylase HENN-1 before being loaded onto the AGO ERGO-1 in the hermaphrodite germline (oocytes) and in embryos (Han et al., 2009; Gent et al., 2010; Vasale et al., 2010; Billi et al., 2012; Kamminga et al., 2012; Montgomery et al., 2012). A separate subset of 26G-RNAs, which is not 3′-methylated, is loaded onto the redundant Argonautes ALG-3, -4 in sperm (Han et al., 2009; Gent et al., 2009; Pavelec et al., 2009; Conine et al., 2010). The ERGO-1 26G-RNA pathway represses expression of genes from repetitive gene families, intergenic and poorly annotated transcripts. In contrast, the ALG-3, -4 pathway mediates both positive and negative regulation of genes required for spermatogenesis and spermiogenesis (Gent et al., 2009, 2010; Han et al., 2009; Pavelec et al., 2009; Conine et al., 2010, 2013; Vasale et al., 2010; Fischer et al., 2011). One general feature of 26G-RNA pathways (like piRNAs, below) is that they trigger the production of secondary 22G-RNAs, which are loaded onto downstream WAGO-type Argonautes to regulate gene expression post-transcriptionally and/or co-transcriptionally.

22G-RNAs are produced by the RdRPs EGO-1 and RRF-1, but in contrast to the 26G-RNAs, do not require Dicer for their biogenesis (Claycomb et al., 2009; Gu et al., 2009). Instead, they are direct products of *de novo* RdRP synthesis (and thus bear a 5′ tri-phosphate). Both EGO-1 and RRF-1 interact with the DEAD-box helicase DRH-3 and the dual Tudor domain protein EKL-1 to form functional RdRP modules (Claycomb et al., 2009; Gu et al., 2009). Two major subsets of 22G-RNAs are parsed between the Argonautes WAGO-1 and CSR-1. While WAGO-1 22G-RNAs are generated by the activity of RRF-1 and EGO-1, and target loci are enriched for repetitive gene families, intergenic regions, and transposable elements, CSR-1 22G-RNAs are generated solely by EGO-1 and target almost exclusively germline-expressed protein coding genes. WAGO-1 has some functional overlap with 11 other WAGOs (Claycomb et al., 2009; Gu et al., 2009; Maniar and Fire, 2011). Consistent with this observation, WAGO-1 partially overlaps in its associated 22G-RNAs and targets with two other WAGOs – WAGO-9/HRDE-1 and NRDE-3 – that function in the nucleus to silence gene expression (Burkhart et al., 2011; Ashe et al., 2012; Buckley et al., 2012; Shirayama et al., 2012). As yet, CSR-1 has not been shown to function redundantly with any other WAGOs. In a departure from the largely negative regulatory roles of known Argonaute pathways, recent studies demonstrate a positive role for CSR-1 in licensing gene expression via nuclear activity. These functions in promoting germline gene expression act in opposition to the silencing activities of the WAGO pathway and piRNAs (Conine et al., 2013; Seth et al., 2013; Wedeles et al., 2013a; Cecere et al., 2014).

**FIGURE 2:**
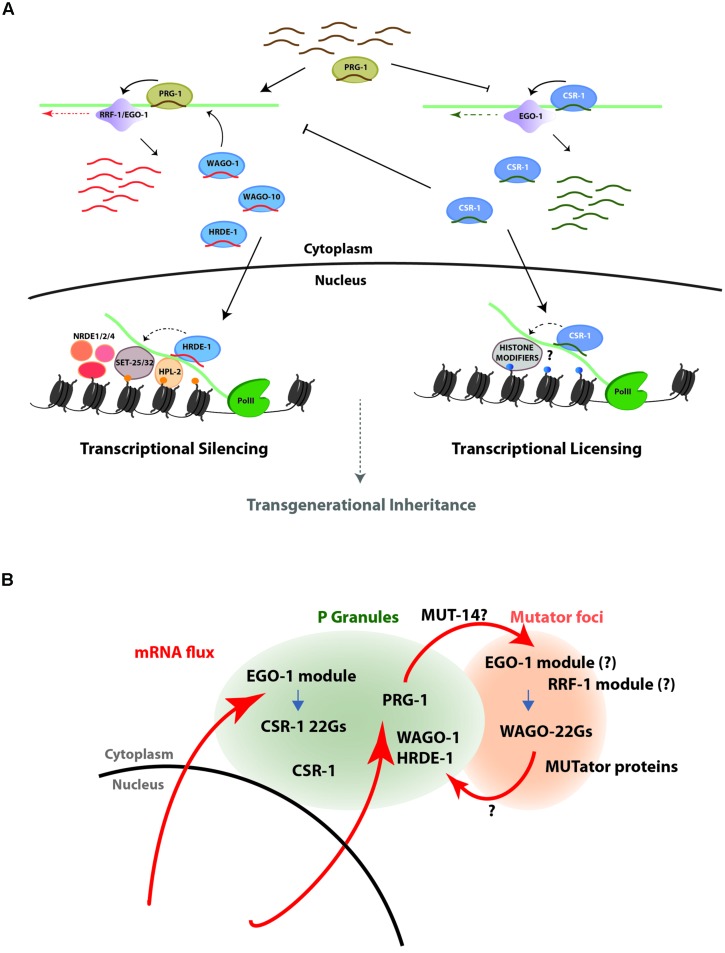
**The PRG-1/WAGO and CSR-1 pathways act in opposition to one another to control the expression state of genes in the *C. elegans* germline. (A)** A schematic representation of the PRG-1/WAGO gene silencing pathway (left) and the CSR-1 gene licensing pathway (right). In the silencing pathway, PRG-1-associated piRNAs trigger production of downstream secondary siRNAs that bind to WAGO-1, which mediates post-transcriptional silencing in the cytoplasm, and to HRDE-1, which translocates to the nucleus to mediate co-transcriptional silencing through inhibition of RNA PolII elongation and transcriptional silencing through deposition of the repressive H3K9me3 mark at target loci. In the licensing pathway, CSR-1 22G-RNA complexes associate with chromatin at target loci and direct a euchromatic state that is permissive for transcription. The pathways appear to be mutually exclusive and self-reinforcing, and at the genetic level each inhibits the activity of the other. **(B)** Schematic representation of a model for germline mRNA flux through perinuclear P granules and nearby Mutator foci, to which many 22G-RNA pathway components localize. The model proposes that germline transcripts pass through P granules as they exit the nuclear pore, where they are scanned for complementarity to CSR-1 and PRG-1/WAGO class 22G-RNAs. Transcripts targeted by CSR-1 are protected from targeting to the WAGO 22G-RNA biosynthesis machinery and thus escape silencing. On the other hand, transcripts not targeted by CSR-1 but with target sites for piRNAs may be transported to Mutator foci for amplification of WAGO-class 22G-RNAs and downstream silencing.

While miRNAs function in various tissues to regulate the timing of expression for key developmental regulators, the majority of small RNA pathways function to protect the *C. elegans* germline genome from foreign or deleterious nucleic acid by silencing. In opposition to this powerful silencing capacity stands the CSR-1 pathway, which promotes expression of its targets in the germline (**Figure 2A**). Targeting by the PRG-1/piRNA pathway and by the CSR-1 pathway are largely mutually exclusive, and these two pathways act in competition with one another to determine the ultimate pattern of gene expression in the germline. Together, silencing (26G-RNAs, piRNAs, WAGO 22G-RNAs) and licensing (CSR-1 22G-RNAs) activities maintain a critical balance of germline gene expression. Furthermore, recent studies indicate roles for these same pathways in reinforcing patterns of gene expression over many generations, by transmitting an epigenetic memory (via small RNAs and histone modifications) to progeny (Ashe et al., 2012; Bagijn et al., 2012; Buckley et al., 2012; Kamminga et al., 2012; Lee et al., 2012; Shirayama et al., 2012; Conine et al., 2013; Seth et al., 2013; Wedeles et al., 2013a; Cecere et al., 2014). In sum, early observations of silencing by miRNAs and exo-RNAi reveal merely the beginning of what has now blossomed into an expanding universe of regulatory roles that are far more complex and interconnected than initially recognized, bringing about many questions regarding pathway cross-talk and specificity.

## CROSS-TALK AND SPECIFICITY

While functional redundancy has been demonstrated among some of the 27 members of the *C. elegans* Argonaute family, it is nonetheless clear that the expansion of Argonaute proteins in the worm has allowed a dramatic diversification of the biological functions carried out by these pathways. A mechanistic understanding of the specificity and functions of these pathways is still in the early stages, but work from a number of labs has begun to address some of the big questions: How are targets selected for each of these pathways? To what extent do these pathways compete for targets and for small RNA binding? What is the biochemical mechanism by which RdRPs synthesize small RNAs of rather precise length? How are the resulting small RNAs sorted onto the appropriate Argonaute – particularly since a number of distinct pathways are housed in the same tissue, the germline? Finally, how do related small RNA:AGO (RISC) complexes carry out diverse downstream functions? We focus here on recent progress toward understanding the questions of how both specificity and diversity of function are accomplished by these related pathways.

Likewise, while a great deal of work remains to be done in the identification and characterization of all of the molecular players in these pathways, a picture is beginning to emerge of a set of strategies that cooperate to achieve both functional diversity and molecular specificity for small RNA pathways in the worm: (1) Spatial and temporal resolution of the expression of AGOs and other pathway factors allows tissue-specific differences in small RNA function; (2) Distinct biochemical properties of the AGOs, their interacting small RNAs, and an incompletely characterized catalog of interacting partners contribute both to the specificity of small RNA sorting and to the diversity of downstream molecular functions. (3) Compartmentalization of small RNA processes into subcellular structures, particularly in the germ lineage, allows for functional specialization among AGOs and 22G-RNAs with overlapping expression patterns.

## DEVELOPMENTAL TIMING OF EXPRESSION AND PATHWAY SPECIFICITY

At the simplest level, some of the differences in the functional outcome in small RNA pathways can be explained by tissue-specific differences in gene expression. For instance, if any of the key components of a small RNA pathway are not expressed in a particular tissue or at a particular point in development, its function simply cannot be mediated. Likewise, due to the nature of RdRP-mediated pathways, which utilize transcripts both as template for small RNA synthesis and as target, the specific profiles of genes expressed in particular tissues may impact which genes are available to be targeted.

While developmentally staged mRNA-seq studies are beginning to shine a light on differences in expression of small RNA pathway components, a comprehensive examination of expression of these factors has yet to be undertaken. Nonetheless, examples of differential AGO expression exist in the literature. For example, the expression profiles of the two 26G-RNA associated AGOs, ALG-3, -4 and ERGO-1, are distinct. While ALG-3, -4 are enriched in stages of development in which spermatogenesis occurs (larval L4 stage, young adult stage, males), ERGO-1 expression is enriched in mature adult hermaphrodites undergoing oogenesis and in embryos (Conine et al., 2010; Vasale et al., 2010). However, differential expression is not sufficient to explain pathway specificity, as most WAGOs and CSR-1 are expressed in the hermaphrodite germline, and both WAGO-1 and CSR-1 are expressed in the male germline as well (Claycomb et al., 2009; Gu et al., 2009; Conine et al., 2010, 2013).

Post-translational modification of AGOs and other pathway components could also contribute to differential pathway activity, as various post-translational modifications of AGOs in other organisms have been shown to regulate AGO function (Meister, 2013). Furthermore, alternative splicing and the existence of multiple AGO isoforms could contribute to differences in binding partners and activity in different tissues. This feature of CSR-1 may be especially critical, as two isoforms of the transcript that differ in their choice of first exon are differentially expressed between males and hermaphrodites (Claycomb et al., 2009; Ortiz et al., 2014). This differential splicing leads to a substantial change in the N-terminus of CSR-1, a portion of the mammalian AGO proteins that has been shown to be critical for endonuclease (slicer) activity toward their target transcripts (Faehnle et al., 2013; Hauptmann et al., 2013; Nakanishi et al., 2013).

## BIOCHEMICAL FEATURES OF SMALL RNAs IMPART SPECIFICITY IN AGO LOADING

The specificity of sorting of small RNAs onto particular AGO proteins was originally studied in the classical miRNA and siRNA pathways. While both siRNAs and miRNAs originate from dsRNA precursors, pre-siRNA duplexes are perfectly paired whereas pre-miRNA duplexes contain characteristic bulges that result from incomplete pairing. Early experiments (Tomari et al., 2007) demonstrated that this characteristic was largely sufficient for the specificity of loading onto RNAi versus miRNA effector Argonautes. In addition, work in plants and flies (Forstemann et al., 2007; Mi et al., 2008) demonstrated that each AGO protein possesses a characteristic preference for the identity of the 5′ nucleotide of the small RNAs to which it binds, and structural studies have since revealed the moieties in the MID domain responsible for this 5′ nucleotide specificity (for example, see Frank et al., 2010). These principles also apply in worms, where the identity of small RNAs bound to a number of AGO proteins can be determined by AGO immunoprecipitation followed by deep sequencing of associated small RNAs, by small RNA high-throughput sequencing to identify small RNAs present at reduced levels in AGO mutants, or by pull-down of a specific small RNA using antisense 2′-*O*-Methylated oligonucleotides followed by immunoblotting to identify the associated AGO protein (Hutvagner et al., 2004; Gu et al., 2011). These methods were used to determine, for example, that the primary (DCR-produced) siRNAs generated during exo-RNAi are associated with RDE-1, while secondary (RdRP-produced) siRNAs are associated with WAGO-family Argonautes (Yigit et al., 2006).

As in other organisms, bulged small RNA duplexes are loaded onto the miRNA effector AGOs ALG-1, -2, while perfect duplexes are loaded onto the RNAi effector RDE-1 (Steiner et al., 2007). Interestingly, the precursor of the miRNA miR-243 has a higher-than-usual degree of complementarity, and a substantial fraction of miR-243 is misloaded onto RDE-1 rather than ALG-1, -2. miR-243 also possesses a 5′ cytosine, whereas most *C. elegans* miRNAs begin with a uracil, consistent with the notion that 5′ nucleotide identity is an important determinant of AGO small RNA specificity (**Figure 1**). Loading specificity has functional consequences, as RDE-1-bound miR-243 functions as a primary siRNA, triggering secondary siRNA production and silencing of a gene that contains a near-perfect complementary target site (Correa et al., 2010).

Perhaps unsurprisingly, unique biochemical properties of worm small RNA classes play an important role in AGO specificity. For example, piRNAs, which possess a 5′ mono-phosphorylated uridine, are bound with high specificity by PRG-1 (Ruby et al., 2006; Batista et al., 2008; Das et al., 2008; Gu et al., 2012). This was particularly evident in studies of the non-canonical piRNAs that are derived from aborted transcripts at canonical gene transcription start sites. Although such aborted transcripts can be detected genome-wide, only those that produce small RNAs with a 5′U are ultimately loaded onto PRG-1 as mature piRNAs (Gu et al., 2012).

Although the specific structural features of worm AGOs that confer specificity to small RNA binding have not yet been identified (no structure for worm AGOs has been solved yet), it is likely that different features of the 5′ and 3′ small RNA binding pockets of the various *C. elegans* AGOs confer some specificity (Yan et al., 2003; Ma et al., 2005; Boland et al., 2010; Elkayam et al., 2012; Schirle and MacRae, 2012). Notably, structure-based alignments of *C. elegans* AGOs with HsAgo2 indicate that a critical tyrosine within the 5′ small RNA binding pocket of human Ago2 (Y529) is absent in many *C. elegans* WAGOs, where it is replaced by a histidine. Y529 is preserved in ALG-1, -2 and RDE-1, which bind to mono-phosphorylated small RNAs (as does HsAgo2). We speculate that this difference and other as-yet unknown differences in the 5′ small RNA binding pocket could contribute to preferences in loading of *C. elegans* AGOs. Clearly, in-depth structure-function studies will be necessary to dissect the features of *C. elegans* AGOs necessary for small RNA binding specificity.

## RdRP MODULES AND COMPARTMENTALIZATION DISTINGUISH 22G-RNA PATHWAYS

It is clear that small RNA properties are not sufficient to explain AGO binding specificity, since CSR-1 and a host of WAGO proteins bind (thus far) biochemically indistinguishable 22G-RNAs that target mostly non-overlapping transcripts (Claycomb et al., 2009; Gu et al., 2009). Specificity for these pathways is particularly important, since they act in opposition to one another. One means of specificity involves using distinct RdRP modules to synthesize 22G-RNAs. As described above, 22G-RNAs of the WAGO pathway are synthesized mainly by RRF-1, with some redundancy provided by EGO-1, while CSR-1 pathway 22G-RNAs are synthesized exclusively by EGO-1. Both RdRPs interact with DRH-3 and EKL-1 to form functional RdRP modules (Claycomb et al., 2009; Gu et al., 2009).

Interestingly, work from several groups has shown that these RdRP modules seem to be localized in adjacent but minimally overlapping perinuclear germline foci. The localization of AGO and PIWI proteins to cytoplasmic ribonucleoprotein foci in the soma and germline is conserved from worms to mammals, and in the germline, such granules possess key functions in germ cell specification, maintenance, and genome surveillance (Voronina et al., 2011). The majority of germline *C. elegans* AGOs examined to date, including CSR-1, WAGO-1, and PRG-1, localize to the perinuclear germline granules of worms, called P granules (Batista et al., 2008; Claycomb et al., 2009; Gu et al., 2009; Updike and Strome, 2010). Notably, expression of CSR-1 is required for wild-type P granule structure; in *csr-1* mutants, P granules detach from the nuclear periphery and collapse into the shared cytoplasm of the worm germline (Claycomb et al., 2009; Updike and Strome, 2009). Furthermore, EGO-1 is also localized to the P granules, suggesting that this is a site of synthesis for the CSR-1 22G-RNAs (Claycomb et al., 2009).

Recent work has identified a distinct set of germline granules in close proximity to the P granules, called Mutator foci (Phillips et al., 2012). Their name derives from the localization to these foci of the mutator class proteins, which are required for small RNA-dependent suppression of transposon mobilization (Ketting et al., 1999). High throughput sequencing of small RNAs from *mutator* strains indicated that mutator proteins are essential for accumulation of WAGO-1 22G-RNAs downstream of PRG-1 and ERGO-1 (Zhang et al., 2011). Thus, Mutator foci are thought to be the sites of biogenesis of this class of small RNAs. This model predicts that other components of the WAGO 22G-RNA machinery, particularly the RRF-1 RdRP module, will localize to Mutator foci as well.

One intriguing component of Mutator foci is the DEAD box RNA helicase MUT-14 (together with its semi-redundant paralog SMUT-1). Although MUT-14 helicase activity is required for the production of 22G-RNAs in the germline, it is dispensable both for production of secondary siRNAs (22G-RNAs) in the soma and for the *in vitro* production of secondary siRNAs. The other mutator proteins are required for 22G-RNA production in all of these scenarios, suggesting that MUT-14 plays a distinct role in the biogenesis pathway. Interestingly, mutator proteins are present diffusely in the cytoplasm of somatic cells, and the compartmentalization normally achieved in the germline by P granules and Mutator foci is not recapitulated under *in vitro* conditions. Based on these observations, the authors suggest that MUT-14 is not directly involved in the biogenesis of 22G-RNAs, but rather in shuttling target transcripts from P granules to Mutator foci for production of WAGO-class 22G-RNAs (Phillips et al., 2014). Lending some support to this idea, the related helicase Vasa in insects has been shown to play a role in transport of piRNA precursor transcripts from the nucleus to the piRNA processing machinery, located in perinuclear germline nuage that is analagous to *C. elegans* P granules (Zhang et al., 2012).

The model that emerges, then, is one where germline transcripts exported through the nuclear pore transit through P granules, where they are scanned for complementarity to CSR-1 and/or PRG-1-bound small RNAs. In this manner, transcripts targeted in P granules by CSR-1 might be distinguished and protected, while PRG-1 targeted transcripts could be identified and shuttled to the Mutator foci. The matter of where WAGO-1 loading ultimately occurs remains unresolved (**Figure 2B**). Although WAGO-1 accumulates in P granules, it is possible that loading occurs during transient occupancy elsewhere (that is not easily detectable by immunofluorescence studies). If this is the case, the compartmentalization of the EGO-1 versus RRF-1 RdRP modules in P granules vs. Mutator foci may play a central role in maintaining specificity of CSR-1 versus WAGO loading. Clarification of the role of subcellular compartmentalization in the specificity of and competition between these two functionally opposing pathways awaits further experiments.

## ROLES FOR SLICER ACTIVITY IN REGULATORY SPECIFICITY?

Slicer activity – the siRNA-dependent endonucleolytic cleavage of target mRNAs – is at the enzymatic center of gene silencing by small RNA pathways. Structural studies of bacterial AGO proteins initially pointed to AGO proteins as possessing the slicer endonuclease activity of the RISC complex (Song et al., 2004), and biochemical experiments quickly confirmed this in eukaryotes (Liu et al., 2004; Meister et al., 2004). Catalysis of RNA cleavage occurs in the RNase H-like active site and requires a conserved DDH catalytic triad (in bacteria) or a DEDD/H catalytic tetrad (in eukaryotes) contained within the PIWI domain present in all AGO proteins (Song et al., 2004; Nakanishi et al., 2012; Swarts et al., 2014). However, not all Argonautes possess slicer activity, and those that do can have very different enzymatic properties that contribute to differences in molecular function (Forstemann et al., 2007).

In *C. elegans*, the catalytic tetrad is absent in the WAGO Argonautes that are the downstream effectors of silencing both for exo-RNAi and for endogenous targets of the WAGO/22G-RNA-pathway (Yigit et al., 2006). Since the WAGO argonautes cannot themselves initiate target degradation by endonucleolytic cleavage, it is presumed that WAGOs recruit an endonuclease to their target mRNAs, although the identity of such protein(s) is unknown. In contrast, the catalytic tetrad is present in CSR-1 and its closest paralog C04F12.1, in PRG-1, in the miRNA Argonautes ALG-1, -2, and in the Argonautes that bind primary siRNAs (RDE-1, ERGO-1, and ALG-3, -4; **Figure 1**). The role of the (in some cases presumed) slicer activity of these Argonautes has been explored for only some of these proteins.

In both flies and humans, the slicer activity of AGO-2 is required both for the endonucleolytic cleavage that initiates degradation of target RNAs and for cleavage of the “passenger” (non-targeting) strand of the siRNA duplex, which facilitates loading of the guide (targeting) strand (Matranga et al., 2005; Miyoshi et al., 2005; Rand et al., 2005). In contrast, the slicer activity of *C. elegans* RDE-1, which binds DCR-1-produced primary exo-siRNAs, appears to be required only for passenger strand removal, with little direct role in target silencing (Steiner et al., 2009). As primary exo-siRNAs alone have little to no silencing activity in *C. elegans* (Sijen et al., 2001; Gu et al., 2009), it is presumed that the ultimate function of RDE-1 and its associated primary siRNAs is to recruit the RdRP module responsible for synthesis of abundant (and potently silencing) secondary siRNAs. It has likewise been suggested, based on an observed increase in 26G-RNA passenger strands in a loss-of-function ERGO-1 mutant, that the catalytic activity of ERGO-1 is required for passenger strand removal (Fischer et al., 2011). It appears possible, then, that for at least a subset of *C. elegans* AGOs, the primary role of slicer activity may be in siRNA biogenesis, rather than in downstream function.

In contrast, ALG-1, -2 have been shown to use slicer activity in the degradation of their target transcripts *in vitro* and *in vivo* (Bouasker and Simard, 2012). For other AGOs with putative catalytic residues, the situation is less clear. While CSR-1 contains the catalytic tetrad and has been shown to be capable of slicing RNAi target mRNAs *in vitro* (Aoki et al., 2007), it is not known whether this activity is required for any of the several CSR-1 functions *in vivo* (see below). Similarly, although PRG-1 has some slicer activity *in vitro* (Bagijn et al., 2012), mutation of either the first or third catalytic aspartic acid residues had little effect on PRG-1 function *in vivo* (Bagijn et al., 2012; Lee et al., 2012). Thus, perhaps unsatisfyingly, the role of slicer activity in regulatory specificity remains a somewhat open question.

## DOWNSTREAM EFFECTORS IMPACT FUNCTIONAL OUTPUTS

Although RNAi was originally described as a post-transcriptional silencing activity, it is now clear that AGOs also direct transcriptional and co-transcriptional silencing in yeast, plants, flies, mammals and worms. In these pathways, Argonaute proteins are known to interact with chromatin modifying factors that direct methylation of histones and/or DNA (in plants and mammals; Castel and Martienssen, 2013). For example, in *Schizosaccharomyces pombe*, an RdRP and Dicer cooperate to generate siRNAs complementary to a number of loci throughout the genome. siRNAs from pericentromeric repetitive loci guide Ago1 to these regions of each chromosome, where Ago1 associates with nascent transcripts. In turn, Ago1 recruits a cascade of chromatin-associated proteins and histone-modifying enzymes, including the histone methyltransferase Clr4, which induces methylation of histone H3 lysine 9 to form heterochromatin in the pericentromeric regions. Strikingly, H3K9 methylation is in turn required for siRNA production, creating a self-reinforcing loop that results in the stable formation of heterochromatin essential for centromere function in *S. pombe* (Goto and Nakayama, 2011). Similarly, it is becoming clear that a complex network of nuclear small RNA pathways plays key roles in both the soma and germline of *C. elegans*.

In somatic tissues of *C. elegans*, the Argonaute NRDE-3 directs exo RNAi-mediated silencing of nuclear transcripts (such as polycistronic operon pre-mRNAs), as well as silencing of some endogenous genes, through a mechanism that involves inhibition of RNA polymerase II elongation and small RNA-dependent trimethylation of Histone H3 at Lysine 9 (H3K9me3; Guang et al., 2010; Burkhart et al., 2011). NRDE-3 contains a bipartite nuclear localization signal that directs 22G-RNA-dependent localization to the nucleus, and nuclear localization is required for the gene regulatory activity of NRDE-3 (Guang et al., 2008). Genetic approaches have begun to reveal the downstream mechanism by which NRDE-3 mediates transcriptional and co-transcriptional silencing, although the mechanistic details remain unclear. Upon import into the nucleus, 22G-bound NRDE-3 associates with nascent target pre-mRNAs and recruits the proteins NRDE-1, NRDE-2, and NRDE-4, which are required for inhibition of RNA Polymerase II elongation. This nuclear pathway likely acts in parallel with canonical post-transcriptional small RNA pathways in the cytoplasm, since targets of the NRDE-3 pathway are derepressed more fully in mutants that lack all secondary siRNAs than they are in *nrde-3* mutants (Guang et al., 2008). RNAi-mediated silencing of somatic targets can be inherited for a single generation in the absence of the RNAi-triggering dsRNA in *C. elegans*, and this inheritance requires nuclear RNAi components to re-establish H3K9me3 in the progeny of RNAi-treated animals (Burton et al., 2011).

In the germline, a mechanistically related nuclear small RNA pathway functions downstream of the PRG-1/piRNA pathway to silence foreign nucleic acids including transgenes, as well as several 100 endogenous loci. This pathway requires the WAGO-clade Argonaute HRDE-1/WAGO-9 (and the semi-redundant Argonaute WAGO-10). HRDE-1 shows 22G-dependent localization to the nucleus, where it associates with the nascent pre-mRNAs of targets and directs H3K9me3 modification (Ashe et al., 2012; Bagijn et al., 2012; Buckley et al., 2012; Kamminga et al., 2012; Lee et al., 2012; Shirayama et al., 2012). Genetic approaches have begun to reveal the downstream mechanisms of HRDE-1-mediated transcriptional silencing. Factors such as NRDE-2 that function in the soma are also required for this germline pathway, as are histone modifiers (such as SET-25, a histone methyltransferase that directs H3K9me3) and chromatin binding proteins (such as HPL-2, the HP1 ortholog in worms, which presumably recognizes H3K9me3; Ashe et al., 2012; Buckley et al., 2012; Shirayama et al., 2012). Strikingly, HRDE-1-dependent chromatin modification and silencing of both foreign and endogenous sequences can be inherited for many generations in the absence of the upstream piRNA trigger in a process termed RNAe (RNA-induced epigenetic silencing).

Like NRDE-3 and HRDE-1, CSR-1 localizes to chromatin at its target loci in a 22G-RNA-dependent manner (Claycomb et al., 2009; Conine et al., 2013). Because CSR-1 interacts with RNA Polymerase II in an RNA-dependent manner, it is likely recruited to target loci through an interaction with nascent transcripts (Wedeles et al., 2013a; Cecere et al., 2014). However, unlike other known nuclear WAGOs, which silence gene expression and promote repressive chromatin modifications, CSR-1 targets are highly enriched for histone modifications associated with euchromatin, including mono- di-, and tri-methylation at histone H3 lysine 4, and acetylation at histone H3 lysine 9, H4 lysine 8, and H4 lysine 16 (van Wolfswinkel and Ketting, 2010; Wedeles et al., 2013b; Cecere et al., 2014). Importantly, recent experiments demonstrate that CSR-1 targeting is sufficient to induce these activating chromatin modifications, since tethering of CSR-1 to a previously WAGO-9-silenced locus is sufficient to activate expression at this locus. Strikingly, after several generations such tethered CSR-1 complexes become loaded with sufficient locus-directed 22G-RNAs to activate expression of a locus with sequence homology in trans, suggesting that CSR-1 22Gs and histone modification participate in a self-reinforcing loop for licensing of germline transcription (Wedeles et al., 2013a).

In support of this model, CSR-1 has been shown to positively regulate the expression of germline genes on a genome-wide scale, and is particularly important during sperm development for promoting the expression of genes involved in sperm differentiation downstream of the ALG-3, -4 26G-RNA pathway (Conine et al., 2013; Cecere et al., 2014). Although the mechanistic details of this spermatogenesis pathway are not yet clear, it seems to be another example of small RNAs acting in a self-reinforcing loop that maintains patterns of gene expression across generations.

Each of these nuclear small RNA pathways in the worm seems to function in a manner with parallels to the role of AGO and chromatin modifiers in pericentromeric heterochromatin formation in *S. pombe*. The similarity between the *C. elegans* HRDE-1 and NRDE-3 pathways and the *S. pombe* centromeric small RNA pathway is evident: small RNAs guide AGO to nascent transcripts, where AGO then recruits chromatin modifying factors that induce heterochromatin formation to silence transcription at the locus. Likewise, it appears that CSR-1 and its associated euchromatin-promoting activities participate in a similar self-reinforcing loop, albeit one that promotes euchromatin domains, and for which a histone modifying component has not yet been identified.

The existence of a gene licensing role for CSR-1, although initially surprising, is consistent with several studies demonstrating that expression of a gene in the germline is required to license expression of that same gene in animals of the subsequent generation (Johnson and Spence, 2011; Gassmann et al., 2012). Both small RNAs and histone modifications have been implicated in this process, and CSR-1-associated 22G-RNAs are likely to serve as a heritable marker of genes expressed in the germline. Likewise, WAGO endo-siRNAs serve as a heritable marker of silencing in previous generations. How, though, is this bi-stable licensed versus silenced state initially set up? Recent experiments indicated that when single-copy transgenes containing non-worm sequences (GFP) are integrated into the *C. elegans* genome, there is some probability that their expression will be silenced in the germline in a PRG-1- and WAGO 22G-RNA-dependent manner. Transgenes that are not silenced are instead targeted by CSR-1 22G-RNAs (Shirayama et al., 2012; Seth et al., 2013). Together, these data suggest a molecular arms race between pathways, with some threshold of targeting being required to tip a target into one pathway or the other. Once a locus is committed to targeting by one pathway or the other, its status is inherited with remarkable fidelity for many generations.

## CONCLUSION

From the elegant simplicity of the discovery of RNAi in *C. elegans*, to the dazzling complexity of the small RNA networks that have emerged in this organism, the worm continues to be at the forefront of small RNA biology. Its early roles in identifying and characterizing the factors, mechanisms, and functional outputs of the miRNA and exo-RNAi pathways were critical in laying a strong foundation for a burgeoning field of gene regulatory biology. Today, work from dozens of labs has greatly expanded our understanding of the number and diversity of *C. elegans* small RNA pathways (Billi et al., 2014). Although several features of *C. elegans* small RNA pathways (particularly the prominent role of RdRPs) appear to be mechanistically distinct from those used in humans or other model animals, it is becoming increasingly clear that the regulatory themes accomplished by these pathways are recurrent.

Protection of germline genome integrity is an intrinsic goal of piRNA pathways in animals. Because the germ lineage is essential for preserving the integrity of a species, it is not surprising that many overlapping regulatory methods act to guard its integrity (Luteijn and Ketting, 2013). Certainly, transposon silencing, as executed by the piRNA pathway in mammals, and mostly by the WAGO 22G-RNA pathway (downstream of the piRNA pathway) in *C. elegans*, acts in a similar manner by silencing at both the transcriptional and post-transcriptional level to defend against the mutagenic effects of transposon mobilization. In addition, piRNA pathway components in insects and mammals are localized to germ granules similar to the P granules and Mutator foci of the worm, and most of the factors in these pathways are required for organismal fertility (Ketting, 2011b; Voronina et al., 2011). In this manner, perhaps some types of endogenous small RNAs in *C. elegans* (WAGO 22G-RNAs, ALG-3, -4 26G-RNAs) that are not formally considered piRNAs are actually functionally equivalent to mammalian piRNAs. To reiterate, although the mechanisms of small RNA biogenesis may diverge between organisms, the functional downstream consequences are recurrent themes.

As we learn more about these pathways, it is likely that additional themes will emerge. For instance, the role of the CSR-1 pathway in licensing rather than silencing germline gene expression came as a surprise to the field, but there are hints from *Drosophila* and mammals that positive gene regulatory roles for Argonautes and small RNA pathways may be a more widespread phenomenon (Li et al., 2006; Cernilogar et al., 2011). The CSR-1 and piRNA pathways also highlight a key regulatory goal for these pathways: maintaining a balance of gene expression (keep “good” genes “on” and “bad” genes “off”), and keeping a record of germline gene expression patterns (likely via small RNAs) to be passed along to the next generation.

In one capacity, these endogenous small RNA pathways could be acting analogously to the miRNA pathway, which is thought to maintain the robustness of gene expression networks in the face of random fluctuation and environmental perturbation (Ebert and Sharp, 2012). Although there appears to be some stochasticity in the initial decision to silence or license a new transcript in the *C. elegans* germline, once this decision is made, it is robustly maintained for many generations by the WAGO or CSR-1 pathways and their downstream chromatin effectors. Conversely, loss of some of these pathways leads to variable phenotypes that could be explained by fluctuations in the expression of their target genes. For example, *alg-3, -4* and *csr-1* mutants display dramatic defects in sperm development that result in complete sterility at 25°C, but relatively wild-type sperm development at 20°C. However, even at 20°C, increasingly penetrant and expressive sterility is observed in *alg-3, -4* populations over the course of several generations. At the molecular level, target genes of ALG-3, -4 and CSR-1 in the male germline fail to maintain expression at 25°C, but are expressed at nearly wild-type levels at 20°C, at least in the initial generation.

How could these phenotypes be explained? It seems plausible that the loss of the ALG-3, -4 pathway may cause an increase in the variability of gene expression from individual to individual that may underlie the incompletely penetrant and progressive sterility observed at 20°C. Such a mechanism would be consistent with observations in a *C. elegans* cell fate specification pathway, where careful measurement of gene expression revealed that mutants with incompletely penetrant phenotypes caused greater variability in the expression of a master regulator transcription factor. Importantly, only some individuals reached the threshold of expression required for wild-type development (Raj et al., 2010). Testing the notion that these small RNA pathways help to buffer changes in gene expression, perhaps even across generational time, will require further careful experimentation. Identifying sources of individual variation in human responses to drugs or susceptibility to disease has been identified as a current challenge where model organisms are likely to make major contributions, and these small RNA pathways certainly fit within that framework (Lehner, 2013).

The worm is an especially attractive context in which to dissect the mechanisms of transgenerational small RNA and epigenetic inheritance. Owing to its genetic and genomic tractability and short generation time, the worm is likely to be at the forefront both of dissecting the molecular mechanisms of transgenerational epigenetic inheritance and of understanding how environmental inputs (including chemical exposure, temperature changes, and nutritional deficiencies) interact with these mechanisms to influence phenotypic variation. In fact, such studies are already underway: a recent study revealed that small RNAs are generated in response to nutritional stresses, and that these small RNAs mediate inheritance of phenotypic responses to the stress for multiple generations (Rechavi et al., 2014). Clearly, the worm’s moment in the small RNA spotlight is not yet over. Both at the level of mechanistic understanding of how small RNA pathways function, and at the level of insight into the biological roles of these pathways, *C. elegans* small RNA research continues to yield important insights.

## Conflict of Interest Statement

The authors declare that the research was conducted in the absence of any commercial or financial relationships that could be construed as a potential conflict of interest.

## References

[B1] Aoki K. Moriguchi H. Yoshioka T. Okawa K. Tabara H. (2007). *In vitro* analyses of the production and activity of secondary small interfering RNAs in *C. elegans*. *EMBO J.* 26 5007–5019 10.1038/sj.emboj.7601910 18007599 PMC2140100

[B2] Ashe A. Sapetschnig A. Weick E. M. Mitchell J. Bagijn M. P. Cording A. C. (2012). piRNAs can trigger a multigenerational epigenetic memory in the germline of *C. elegans*. *Cell* 150 88–99 10.1016/j.cell.2012.06.018 22738725 PMC3464430

[B3] Bagijn M. P. Goldstein L. D. Sapetschnig A. Weick E. M. Bouasker S. Lehrbach N. J. (2012). Function, targets, and evolution of *Caenorhabditis elegans* piRNAs. *Science* 337 574–578 10.1126/science.1220952 22700655 PMC3951736

[B4] Batista P. J. Ruby J. G. Claycomb J. M. Chiang R. Fahlgren N. Kasschau K. D. (2008). PRG-1 and 21U-RNAs interact to form the piRNA complex required for fertility in *C. elegans*. *Mol. Cell* 31 67–78 10.1016/j.molcel.2008.06.002 18571452 PMC2570341

[B5] Bernstein E. Caudy A. A. Hammond S. M. Hannon G. J. (2001). Role for a bidentate ribonuclease in the initiation step of RNA interference. *Nature* 409 363–366 10.1038/35053110 11201747

[B6] Billi A. C. Alessi A. F. Khivansara V. Han T. Freeberg M. Mitani S. (2012). The *Caenorhabditis elegans* HEN1 Ortholog, HENN-1, methylates and stabilizes select subclasses of germline small RNAs. *PLoS Genet.* 8:e1002617 10.1371/journal.pgen.1002617 PMC3330095 22548001

[B7] Billi A. C. Fischer S. E. Kim J. K. (2014). Endogenous RNAi pathways in *C. elegans*. *WormBook* 1–49 10.1895/wormbook.1.170.1 24816713 PMC4781133

[B8] Billi A. C. Freeberg M. A. Day A. M. Chun S. Y. Khivansara V. Kim J. K. (2013). A conserved upstream motif orchestrates autonomous, germline-enriched expression of *Caenorhabditis elegans* piRNAs. *PLoS Genet.* 9:e1003392 10.1371/journal.pgen.1003392 PMC3597512 23516384

[B9] Boland A. Tritschler F. Heimstadt S. Izaurralde E. Weichenrieder O. (2010). Crystal structure and ligand binding of the MID domain of a eukaryotic Argonaute protein. *EMBO Rep.* 11 522–527 10.1038/embor.2010.81 20539312 PMC2897117

[B10] Bouasker S. Simard M. J. (2012). The slicing activity of miRNA-specific Argonautes is essential for the miRNA pathway in *C. elegans.* *Nucleic Acids Res.* 40 10452–10462 10.1093/nar/gks748 22904066 PMC3488219

[B11] Brenner S. (1974). The genetics of *Caenorhabditis elegans*. *Genetics* 77 71–94. 4366476 10.1093/genetics/77.1.71 PMC1213120

[B12] Buckley B. A. Burkhart K. B. Gu S. G. Spracklin G. Kershner A. Fritz H. (2012). A nuclear Argonaute promotes multigenerational epigenetic inheritance and germline immortality. *Nature* 489 447–451 10.1038/nature11352 22810588 PMC3509936

[B13] Burkhart K. B. Guang S. Buckley B. A. Wong L. Bochner A. F. Kennedy S. (2011). A Pre-mRNA-associating factor links endogenous siRNAs to chromatin regulation. *PLoS Genet.* 7:e1002249 10.1371/journal.pgen.1002249 PMC3161925 21901112

[B14] Burton N. O. Burkhart K. B. Kennedy S. (2011). Nuclear RNAi maintains heritable gene silencing in *Caenorhabditis elegans*. *Proc. Natl. Acad. Sci. U.S.A.* 108 19683–19688 10.1073/pnas.1113310108 22106253 PMC3241819

[B15] Castel S. E. Martienssen R. A. (2013). RNA interference in the nucleus: roles for small RNAs in transcription, epigenetics and beyond. *Nat. Rev. Genet.* 14 100–112 10.1038/nrg3355 23329111 PMC4205957

[B16] Cecere G. Hoersch S. O’Keeffe S. Sachidanandam R. Grishok A. (2014). Global effects of the CSR-1 RNA interference pathway on the transcriptional landscape. *Nat. Struct. Mol. Biol.* 21 358–365 10.1038/nsmb.2801 24681887 PMC4068146

[B17] Cernilogar F. M. Onorati M. C. Kothe G. O. Burroughs A. M. Parsi K. M. Breiling A. (2011). Chromatin-associated RNA interference components contribute to transcriptional regulation in *Drosophila*. *Nature* 480 391–395 10.1038/nature10492 22056986 PMC4082306

[B18] Claycomb J. M. Batista P. J. Pang K. M. Gu W. Vasale J. J. van Wolfswinkel J. C. (2009). The Argonaute CSR-1 and its 22G-RNA cofactors are required for holocentric chromosome segregation. *Cell* 139 123–134 10.1016/j.cell.2009.09.014 19804758 PMC2766185

[B19] Conine C. C. Batista P. J. Gu W. Claycomb J. M. Chaves D. A. Shirayama M. (2010). Argonautes ALG-3 and ALG-4 are required for spermatogenesis-specific 26G-RNAs and thermotolerant sperm in *Caenorhabditis elegans*. *Proc. Natl. Acad. Sci. U.S.A.* 107 3588–3593 10.1073/pnas.0911685107 20133686 PMC2840486

[B20] Conine C. C. Moresco J. J. Gu W. Shirayama M. Conte D. J. Yates J. R. R. (2013). Argonautes promote male fertility and provide a paternal memory of germline gene expression in *C. elegans*. *Cell* 155 1532–1544 10.1016/j.cell.2013.11.032 24360276 PMC3924572

[B21] Correa R. L. Steiner F. A. Berezikov E. Ketting R. F. (2010). MicroRNA-directed siRNA biogenesis in *Caenorhabditis elegans*. *PLoS Genet.* 6:e1000903 10.1371/journal.pgen.1000903 PMC2851571 20386745

[B22] Czech B. Hannon G. J. (2011). Small RNA sorting: matchmaking for Argonautes. *Nat. Rev. Genet.* 12 19–31 10.1038/nrg2916 21116305 PMC3703915

[B23] Das P. P. Bagijn M. P. Goldstein L. D. Woolford J. R. Lehrbach N. J. Sapetschnig A. (2008). Piwi and piRNAs act upstream of an endogenous siRNA pathway to suppress Tc3 transposon mobility in the *Caenorhabditis elegans* germline. *Mol. Cell* 31 79–90 10.1016/j.molcel.2008.06.003 18571451 PMC3353317

[B24] Duchaine T. F. Wohlschlegel J. A. Kennedy S. Bei Y. Conte D. J. Pang K. (2006). Functional proteomics reveals the biochemical niche of *C. elegans* DCR-1 in multiple small-RNA-mediated pathways. *Cell* 124 343–354 10.1016/j.cell.2005.11.036 16439208

[B25] Ebert M. S. Sharp P. A. (2012). Roles for microRNAs in conferring robustness to biological processes. *Cell* 149 515–524 10.1016/j.cell.2012.04.005 22541426 PMC3351105

[B26] Elkayam E. Kuhn C. D. Tocilj A. Haase A. D. Greene E. M. Hannon G. J. (2012). The structure of human argonaute-2 in complex with miR-20a. *Cell* 150 100–110 10.1016/j.cell.2012.05.017 22682761 PMC3464090

[B27] Faehnle C. R. Elkayam E. Haase A. D. Hannon G. J. Joshua-Tor L. (2013). The making of a slicer: activation of human Argonaute-1. *Cell Rep.* 3 1901–1909 10.1016/j.celrep.2013.05.033 23746446 PMC3769929

[B28] Fire A. Xu S. Montgomery M. K. Kostas S. A. Driver S. E. Mello C. C. (1998). Potent and specific genetic interference by double-stranded RNA in *Caenorhabditis elegans*. *Nature* 391 806–811 10.1038/35888 9486653

[B29] Fischer S. E. Montgomery T. A. Zhang C. Fahlgren N. Breen P. C. Hwang A. (2011). The ERI-6/7 helicase acts at the first stage of an siRNA amplification pathway that targets recent gene duplications. *PLoS Genet.* 7:e1002369 10.1371/journal.pgen.1002369 PMC3213143 22102828

[B30] Forstemann K. Horwich M. D. Wee L. Tomari Y. Zamore P. D. (2007). *Drosophila* microRNAs are sorted into functionally distinct argonaute complexes after production by dicer-1. *Cell* 130 287–297 10.1016/j.cell.2007.05.056 17662943 PMC2686109

[B31] Frank F. Sonenberg N. Nagar B. (2010). Structural basis for 5′-nucleotide base-specific recognition of guide RNA by human AGO2. *Nature* 465 818–822 10.1038/nature09039 20505670

[B32] Gassmann R. Rechtsteiner A. Yuen K. W. Muroyama A. Egelhofer T. Gaydos L. (2012). An inverse relationship to germline transcription defines centromeric chromatin in *C. elegans*. *Nature* 484 534–537 10.1038/nature10973 22495302 PMC3538161

[B33] Gent J. I. Lamm A. T. Pavelec D. M. Maniar J. M. Parameswaran P. Tao L. (2010). Distinct phases of siRNA synthesis in an endogenous RNAi pathway in *C. elegans* soma. *Mol. Cell.* 37 679–689 10.1016/j.molcel.2010.01.012 20116306 PMC2838994

[B34] Gent J. I. Schvarzstein M. Villeneuve A. M. Gu S. G. Jantsch V. Fire A. Z. (2009). A *Caenorhabditis elegans* RNA-directed RNA polymerase in sperm development and endogenous RNA interference. *Genetics* 183 1297–1314 10.1534/genetics.109.109686 19805814 PMC2787422

[B35] Goto D. B. Nakayama J. I. (2011). RNA and epigenetic silencing: insight from fission yeast. *Dev. Growth Differ.* 54 129–141 10.1111/j.1440-169X.2011.01310.x 22150237 PMC3380556

[B36] Gu W. Claycomb J. M. Batista P. J. Mello C. C. Conte D. (2011). Cloning Argonaute-associated small RNAs from *Caenorhabditis elegans*. *Methods Mol. Biol.* 725 251–280 10.1007/978-1-61779-046-1_17 21528459

[B37] Gu W. Lee H. C. Chaves D. Youngman E. M. Pazour G. J. Conte D. J. (2012). CapSeq and CIP-TAP identify Pol II start sites and reveal capped small RNAs as *C. elegans* piRNA precursors. *Cell* 151 1488–1500 10.1016/j.cell.2012.11.023 23260138 PMC3581324

[B38] Gu W. Shirayama M. Conte D. J. Vasale J. Batista P. J. Claycomb J. M. (2009). Distinct argonaute-mediated 22G-RNA pathways direct genome surveillance in the *C. elegans* germline. *Mol. Cell* 36 231–244 10.1016/j.molcel.2009.09.020 19800275 PMC2776052

[B39] Guang S. Bochner A. F. Burkhart K. B. Burton N. Pavelec D. M. Kennedy S. (2010). Small regulatory RNAs inhibit RNA polymerase II during the elongation phase of transcription. *Nature* 465 1097–1101 10.1038/nature09095 20543824 PMC2892551

[B40] Guang S. Bochner A. F. Pavelec D. M. Burkhart K. B. Harding S. Lachowiec J. (2008). An Argonaute transports siRNAs from the cytoplasm to the nucleus. *Science* 321 537–541 10.1126/science.1157647 18653886 PMC2771369

[B41] Guo S. Kemphues K. J. (1995). par-1, a gene required for establishing polarity in *C. elegans* embryos, encodes a putative Ser/Thr kinase that is asymmetrically distributed. *Cell* 81 611–620 10.1016/0092-8674(95)90082-9 7758115

[B42] Han T. Manoharan A. P. Harkins T. T. Bouffard P. Fitzpatrick C. Chu D. S. (2009). 26G endo-siRNAs regulate spermatogenic and zygotic gene expression in *Caenorhabditis elegans*. *Proc. Natl. Acad. Sci. U.S.A.* 106 18674–18679 10.1073/pnas.0906378106 19846761 PMC2765456

[B43] Hauptmann J. Dueck A. Harlander S. Pfaff J. Merkl R. Meister G. (2013). Turning catalytically inactive human Argonaute proteins into active slicer enzymes. *Nat. Struct. Mol. Biol.* 20 814–817 10.1038/nsmb.2577 23665583

[B44] Hutvagner G. Simard M. J. (2008). Argonaute proteins: key players in RNA silencing. *Nat. Rev. Mol. Cell Biol.* 9 22–32 10.1038/nrm2321 18073770

[B45] Hutvagner G. Simard M. J. Mello C. C. Zamore P. D. (2004). Sequence-specific inhibition of small RNA function. *PLoS Biol.* 2:E98 10.1371/journal.pbio.0020098 PMC350664 15024405

[B46] Johnson C. L. Spence A. M. (2011). Epigenetic licensing of germline gene expression by maternal RNA in *C. elegans*. *Science* 333 1311–1314 10.1126/science.1208178 21885785

[B47] Kamath R. S. Fraser A. G. Dong Y. Poulin G. Durbin R. Gotta M. (2003). Systematic functional analysis of the *Caenorhabditis elegans* genome using RNAi. *Nature* 421 231–237 10.1038/nature01278 12529635

[B48] Kamminga L. M. van Wolfswinkel J. C. Luteijn M. J. Kaaij L. J. Bagijn M. P. Sapetschnig A. (2012). Differential Impact of the HEN1 Homolog HENN-1 on 21U and 26G RNAs in the Germline of *Caenorhabditis elegans*. *PLoS Genet.* 8:e1002702 10.1371/journal.pgen.1002702 PMC3400576 22829772

[B49] Ketting R. F. (2011a). microRNA biogenesis and function : an overview. *Adv. Exp. Med. Biol.* 700 1–14 10.1007/978-1-4419-7823-3_1 21755468

[B50] Ketting R. F. (2011b). The many faces of RNAi. *Dev. Cell* 20 148–161 10.1016/j.devcel.2011.01.012 21316584

[B51] Ketting R. F. Fischer S. E. Bernstein E. Sijen T. Hannon G. J. Plasterk R. H. (2001). Dicer functions in RNA interference and in synthesis of small RNA involved in developmental timing in *C. elegans*. *Genes Dev.* 15 2654–2659 10.1101/gad.927801 11641272 PMC312808

[B52] Ketting R. F. Haverkamp T. H. van Luenen H. G. Plasterk R. H. (1999). Mut-7 of *C. elegans*, required for transposon silencing and RNA interference, is a homolog of Werner syndrome helicase and RNaseD. *Cell* 99 133–141 10.1016/S0092-8674(00)81645-1 10535732

[B53] Knight S. W. Bass B. L. (2001). A role for the RNase III enzyme DCR-1 in RNA interference and germ line development in *Caenorhabditis elegans*. *Science* 293 2269–2271 10.1126/science.1062039 11486053 PMC1855227

[B54] Lagos-Quintana M. Rauhut R. Lendeckel W. Tuschl T. (2001). Identification of novel genes coding for small expressed RNAs. *Science* 294 853–858 10.1126/science.1064921 11679670

[B55] Lau N. C. Lim L. P. Weinstein E. G. Bartel D. P. (2001). An abundant class of tiny RNAs with probable regulatory roles in *Caenorhabditis elegans*. *Science* 294 858–862 10.1126/science.1065062 11679671

[B56] Lee H. C. Gu W. Shirayama M. Youngman E. Conte D. J. Mello C. C. (2012). *C. elegans* piRNAs mediate the genome-wide surveillance of germline transcripts. *Cell* 150 78–87 10.1016/j.cell.2012.06.016 22738724 PMC3410639

[B57] Lee R. C. Ambros V. (2001). An extensive class of small RNAs in *Caenorhabditis elegans*. *Science* 294 862–864 10.1126/science.1065329 11679672

[B58] Lee R. C. Feinbaum R. L. Ambros V. (1993). The *C. elegans* heterochronic gene lin-4 encodes small RNAs with antisense complementarity to lin-14. *Cell* 75 843–854 10.1016/0092-8674(93)90529-Y 8252621

[B59] Lehner B. (2013). Genotype to phenotype: lessons from model organisms for human genetics. *Nat. Rev. Genet.* 14 168–178 10.1038/nrg3404 23358379

[B60] Li L. C. Okino S. T. Zhao H. Pookot D. Place R. F. Urakami S. (2006). Small dsRNAs induce transcriptional activation in human cells. *Proc. Natl. Acad. Sci. U.S.A.* 103 17337–17342 10.1073/pnas.0607015103 17085592 PMC1859931

[B61] Liu J. Carmell M. A. Rivas F. V. Marsden C. G. Thomson J. M. Song J. J. (2004). Argonaute2 is the catalytic engine of mammalian RNAi. *Science* 305 1437–1441 10.1126/science.1102513 15284456

[B62] Luteijn M. J. Ketting R. F. (2013). PIWI-interacting RNAs: from generation to transgenerational epigenetics. *Nat. Rev. Genet.* 14 523–534 10.1038/nrg3495 23797853

[B63] Ma J. B. Yuan Y. R. Meister G. Pei Y. Tuschl T. Patel D. J. (2005). Structural basis for 5′-end-specific recognition of guide RNA by the *A. fulgidus* Piwi protein. *Nature* 434 666–670 10.1038/nature03514 15800629 PMC4694588

[B64] Maniar J. M. Fire A. Z. (2011). EGO-1, a *C. elegans* RdRP, modulates gene expression via production of mRNA-templated short antisense RNAs. *Curr. Biol.* 21 449–459 10.1016/j.cub.2011.02.019 21396820 PMC3073447

[B65] Martinez J. Patkaniowska A. Urlaub H. Luhrmann R. Tuschl T. (2002). Single-stranded antisense siRNAs guide target RNA cleavage in RNAi. *Cell* 110 563–574 10.1016/S0092-8674(02)00908-X 12230974

[B66] Matranga C. Tomari Y. Shin C. Bartel D. P. Zamore P. D. (2005). Passenger-strand cleavage facilitates assembly of siRNA into Ago2-containing RNAi enzyme complexes. *Cell* 123 607–620 10.1016/j.cell.2005.08.044 16271386

[B67] Meister G. (2013). Argonaute proteins: functional insights and emerging roles. *Nat. Rev. Genet.* 14 447–459 10.1038/nrg3462 23732335

[B68] Meister G. Landthaler M. Patkaniowska A. Dorsett Y. Teng G. Tuschl T. (2004). Human argonaute2 mediates RNA cleavage targeted by miRNAs and siRNAs. *Mol. Cell* 15 185–197 10.1016/j.molcel.2004.07.007 15260970

[B69] Mi S. Cai T. Hu Y. Chen Y. Hodges E. Ni F. (2008). Sorting of small RNAs into Arabidopsis argonaute complexes is directed by the 5′ terminal nucleotide. *Cell* 133 116–127 10.1016/j.cell.2008.02.034 18342361 PMC2981139

[B70] Miyoshi K. Tsukumo H. Nagami T. Siomi H. Siomi M. C. (2005). Slicer function of *Drosophila* Argonautes and its involvement in RISC formation. *Genes Dev.* 19 2837–2848 10.1101/gad.1370605 16287716 PMC1315391

[B71] Moffat J. Grueneberg D. A. Yang X. Kim S. Y. Kloepfer A. M. Hinkle G. (2006). A lentiviral RNAi library for human and mouse genes applied to an arrayed viral high-content screen. *Cell* 124 1283–1298 10.1016/j.cell.2006.01.040 16564017

[B72] Montgomery T. A. Rim Y. S. Zhang C. Dowen R. H. Phillips C. M. Fischer S. E. (2012). PIWI associated siRNAs and piRNAs specifically require the *Caenorhabditis elegans* HEN1 ortholog henn-1. *PLoS Genet.* 8:e1002616 10.1371/journal.pgen.1002616 PMC3334881 22536158

[B73] Nakanishi K. Ascano M. Gogakos T. Ishibe-Murakami S. Serganov A. A. Briskin D. (2013). Eukaryote-specific insertion elements control human ARGONAUTE slicer activity. *Cell Rep.* 3 1893–1900 10.1016/j.celrep.2013.06.010 23809764 PMC3757560

[B74] Nakanishi K. Weinberg D. E. Bartel D. P. Patel D. J. (2012). Structure of yeast Argonaute with guide RNA. *Nature* 486 368–374 10.1038/nature11211 22722195 PMC3853139

[B75] Nuez I. Felix M. A. (2012). Evolution of susceptibility to ingested double-stranded RNAs in *Caenorhabditis* nematodes. *PLoS ONE* 7:e29811 10.1371/journal.pone.0029811 PMC3256175 22253787

[B76] Ortiz M. A. Noble D. Sorokin E. P. Kimble J. (2014). A new dataset of spermatogenic vs. oogenic transcriptomes in the nematode *Caenorhabditis elegans*. *G3 (Bethesda)* 4 1765–1772 10.1534/g3.114.012351 25060624 PMC4169169

[B77] Pavelec D. M. Lachowiec J. Duchaine T. F. Smith H. E. Kennedy S. (2009). Requirement for the ERI/DICER complex in endogenous RNA interference and sperm development in *Caenorhabditis elegans*. *Genetics* 183 1283–1295 10.1534/genetics.109.108134 19797044 PMC2787421

[B78] Phillips C. M. Montgomery B. E. Breen P. C. Roovers E. F. Rim Y. S. Ohsumi T. K. (2014). MUT-14 and SMUT-1 DEAD box RNA helicases have overlapping roles in germline RNAi and endogenous siRNA formation. *Curr. Biol.* 24 839–844 10.1016/j.cub.2014.02.060 24684932 PMC4010136

[B79] Phillips C. M. Montgomery T. A. Breen P. C. Ruvkun G. (2012). MUT-16 promotes formation of perinuclear mutator foci required for RNA silencing in the *C. elegans* germline. *Genes Dev.* 26 1433–1444 10.1101/gad.193904.112 22713602 PMC3403012

[B80] Raj A. Rifkin S. A. Andersen E. van Oudenaarden A. (2010). Variability in gene expression underlies incomplete penetrance. *Nature* 463 913–918 10.1038/nature08781 20164922 PMC2836165

[B81] Rand T. A. Petersen S. Du F. Wang X. (2005). Argonaute2 cleaves the anti-guide strand of siRNA during RISC activation. *Cell* 123 621–629 10.1016/j.cell.2005.10.020 16271385

[B82] Rechavi O. Houri-Ze’evi L. Anava S. Goh W. S. Kerk S. Y. Hannon G. J. (2014). Starvation-induced transgenerational inheritance of small RNAs in *C. elegans*. *Cell* 158 277–287 10.1016/j.cell.2014.06.020 25018105 PMC4377509

[B83] Riddle D. L. Blumenthal T. Meyer B. J. Priess J. R. (1997). “Introduction to *C. elegans*,” in *C. elegans II* eds Riddle D. L. Blumenthal T. Meyer B. J. Priess J. R. Priess (Cold Spring Harbor, NY: SHL) 1–22.

[B84] Rocheleau C. E. Downs W. D. Lin R. Wittmann C. Bei Y. Cha Y. H. (1997). Wnt signaling and an APC-related gene specify endoderm in early *C. elegans* embryos. *Cell* 90 707–716 10.1016/S0092-8674(00)80531-0 9288750

[B85] Ruby J. G. Jan C. Player C. Axtell M. J. Lee W. Nusbaum C. (2006). Large-scale sequencing reveals 21U-RNAs and additional microRNAs and endogenous siRNAs in *C. elegans*. *Cell* 127 1193–1207 10.1016/j.cell.2006.10.040 17174894

[B86] Schirle N. T. MacRae I. J. (2012). The crystal structure of human Argonaute2. *Science* 336 1037–1040 10.1126/science.1221551 22539551 PMC3521581

[B87] Seth M. Shirayama M. Gu W. Ishidate T. Conte D. J. Mello C. C. (2013). The *C. elegans* CSR-1 argonaute pathway counteracts epigenetic silencing to promote germline gene expression. *Dev. Cell* 27 656–663 10.1016/j.devcel.2013.11.014 24360782 PMC3954781

[B88] Shirayama M. Seth M. Lee H. C. Gu W. Ishidate T. Conte D. J. (2012). piRNAs initiate an epigenetic memory of nonself RNA in the *C. elegans* germline. *Cell* 150 65–77 10.1016/j.cell.2012.06.015 22738726 PMC3597741

[B89] Sijen T. Fleenor J. Simmer F. Thijssen K. L. Parrish S. Timmons L. (2001). On the role of RNA amplification in dsRNA-triggered gene silencing. Cell 107 465–476 10.1016/S0092-8674(01)00576-1 11719187

[B90] Smardon A. Spoerke J. M. Stacey S. C. Klein M. E. Mackin N. Maine E. M. (2000). EGO-1 is related to RNA-directed RNA polymerase and functions in germ-line development and RNA interference in *C. elegans.* *Curr. Biol.* 10 169–178 10.1016/S0960-9822(00)00323-7 10704412

[B91] Song J. J. Smith S. K. Hannon G. J. Joshua-Tor L. (2004). Crystal structure of Argonaute and its implications for RISC slicer activity. *Science* 305 1434–1437 10.1126/science.1102514 15284453

[B92] Steiner F. A. Hoogstrate S. W. Okihara K. L. Thijssen K. L. Ketting R. F. Plasterk R. H. (2007). Structural features of small RNA precursors determine Argonaute loading in *Caenorhabditis elegans*. *Nat. Struct. Mol. Biol.* 14 927–933 10.1038/nsmb1308 17891148

[B93] Steiner F. A. Okihara K. L. Hoogstrate S. W. Sijen T. Ketting R. F. (2009). RDE-1 slicer activity is required only for passenger-strand cleavage during RNAi in *Caenorhabditis elegans*. *Nat. Struct. Mol. Biol.* 16 207–211 10.1038/nsmb.1541 19151723

[B94] Swarts D. C. Makarova K. Wang Y. Nakanishi K. Ketting R. F. Koonin E. V. (2014). The evolutionary journey of Argonaute proteins. *Nat. Struct. Mol. Biol.* 21 743–753 10.1038/nsmb.2879 25192263 PMC4691850

[B95] Tabara H. Sarkissian M. Kelly W. G. Fleenor J. Grishok A. Timmons L. (1999). The rde-1 gene, RNA interference, and transposon silencing in *C. elegans*. *Cell* 99 123–132 10.1016/S0092-8674(00)81644-X 10535731

[B96] Tomari Y. Du T. Zamore P. D. (2007). Sorting of *Drosophila* small silencing RNAs. *Cell* 130 299–308 10.1016/j.cell.2007.05.057 17662944 PMC2841505

[B97] Updike D. Strome S. (2010). P granule assembly and function in *Caenorhabditis elegans* germ cells. *J. Androl*. 31 53–60 10.2164/jandrol.109.008292 19875490 PMC2905540

[B98] Updike D. L. Strome S. (2009). A genomewide RNAi screen for genes that affect the stability, distribution and function of P granules in *Caenorhabditis elegans*. *Genetics* 183 1397–1419 10.1534/genetics.109.110171 19805813 PMC2787428

[B99] van Wolfswinkel J. C. Ketting R. F. (2010). The role of small non-coding RNAs in genome stability and chromatin organization. *J. Cell Sci.* 123 1825–1839 10.1242/jcs.061713 20484663

[B100] Vasale J. J. Gu W. Thivierge C. Batista P. J. Claycomb J. M. Youngman E. M. (2010). Sequential rounds of RNA-dependent RNA transcription drive endogenous small-RNA biogenesis in the ERGO-1/Argonaute pathway. *Proc. Natl. Acad. Sci. U.S.A.* 107 3582–3587 10.1073/pnas.0911908107 20133583 PMC2840456

[B101] Voronina E. Seydoux G. Sassone-Corsi P. Nagamori I. (2011). RNA granules in germ cells. *Cold Spring Harb. Perspect. Biol.* 3 pii: a002774 10.1101/cshperspect.a002774 PMC3225947 21768607

[B102] Wang G. Reinke V. (2008). A *C. elegans* Piwi, PRG-1, regulates 21U-RNAs during spermatogenesis. *Curr. Biol.* 18 861–867 10.1016/j.cub.2008.05.009 18501605 PMC2494713

[B103] Wedeles C. J. Wu M. Z. Claycomb J. M. (2013a). Protection of germline gene expression by the *C. elegans* Argonaute CSR-1. *Dev. Cell* 27 664–671 10.1016/j.devcel.2013.11.016 24360783

[B104] Wedeles C. J. Wu M. Z. Claycomb J. M. (2013b). A multitasking Argonaute: exploring the many facets of *C. elegans* CSR-1. *Chromosome Res.* 1 573–586 10.1007/s10577-013-9383-7 24178449

[B105] Whangbo J. S. Hunter C. P. (2008). Environmental RNA interference. *Trends Genet.* 24 297–305 10.1016/j.tig.2008.03.007 18450316

[B106] Wightman B. Ha I. Ruvkun G. (1993). Posttranscriptional regulation of the heterochronic gene lin-14 by lin-4 mediates temporal pattern formation in *C. elegans*. *Cell* 75 855–862 10.1016/0092-8674(93)90530-4 8252622

[B107] Winston W. M. Sutherlin M. Wright A. J. Feinberg E. H. Hunter C. P. (2007). Caenorhabditis elegans SID-2 is required for environmental RNA interference. *Proc. Natl. Acad. Sci. U.S.A.* 104 10565–10570 10.1073/pnas.0611282104 17563372 PMC1965553

[B108] Yan K. S. Yan S. Farooq A. Han A. Zeng L. Zhou M. M. (2003). Structure and conserved RNA binding of the PAZ domain. *Nature* 426 468–474 10.1038/nature02129 14615802

[B109] Yigit E. Batista P. J. Bei Y. Pang K. M. Chen C. C. Tolia N. H. (2006). Analysis of the *C. elegans* Argonaute family reveals that distinct Argonautes act sequentially during RNAi. *Cell* 127 747–757 10.1016/j.cell.2006.09.033 17110334

[B110] Zhang C. Montgomery T. A. Gabel H. W. Fischer S. E. Phillips C. M. Fahlgren N. (2011). mut-16 and other mutator class genes modulate 22G and 26G siRNA pathways in *Caenorhabditis elegans*. *Proc. Natl. Acad. Sci. U.S.A.* 108 1201–1208 10.1073/pnas.1018695108 21245313 PMC3029761

[B111] Zhang F. Wang J. Xu J. Zhang Z. Koppetsch B. S. Schultz N. (2012). UAP56 couples piRNA clusters to the perinuclear transposon silencing machinery. *Cell* 151 871–884 10.1016/j.cell.2012.09.040 23141543 PMC3499805

